# Supplementary material for: What Is Hidden in Patients with Unknown Nephropathy? Genetic Screening Could Be the Missing Link in Kidney Transplantation Diagnosis and Management
Source: Int J Mol Sci. 2024 Jan 24;25(3):1436. doi: 10.3390/ijms25031436 (PMC10855929; doi:10.3390/ijms25031436)
Supplement: Supplementary file 1 [file ijms-25-01436-s001.zip › ijms-2811632-supplementary.pdf]

Supplementary Methods

S1: Genes included in the NGS-custom panel

ACTN4 (NM_004924.6),ADCK4 (NM_024876.4), ALG1 (NM_019109.5), ANLN (NM_018685.5), APOL1 (NM_001136540.1), ARHGAP24 (NM_001025616.3), ARHGDIA (NM_004309.6), CD151 (NM_001039490.1), CD2AP (NM_012120.3), CFH (NM_000186.4), CLCN5 (NM_001127898.4), COL4A3 (NM_000091.5), COL4A4 (NM_000092.5), COL4A5 (NM_000495.5), COQ2 (NM_015697.8), COQ6 (NM_182476.3), CRB2 (NM_173689.7), CTNS (NM_001031681.2), CUBN (NM_001081.4), DGKE (NM_003647.3), E2F3 (NM_001949.5), EMP2 (NM_001424.6), EYA1 (NM_172058.4), INF2 (NM_000268.3),ITGA3 (NM_002204.4), ITGB4 (NM_000213.5), KANK1 (NM_015158.4), KANK2 (NM_015493.6), KANK4 (NM_181712.5), KL (NM_004795.4), LAMB2 (NM_002292), LCAT (NM_000229.2), LMN (NM_170707.4), LMX1B (NM_001174147.2), MYH9 (NM_002473.5), MYO1E (NM_004998.4),NUP93 (NM_014669.5), NUP107 (NM_020401.4), NUP205 (NM_015135.3), NPHS1 (NM_004646.3), NPHS2 (NM_014625.4), NXF5 (NM_032946.2), OCRL (NM_000276.4), PAX2 (NM_003990.5), PDSS2 (NM_020381.4), PLCE1 (NM_016341.4), PMM2 (NM_000303.3), PODXL (NM_005397.4), PTPRO (NM_030667.3), SCARB2 (NM_005506.4), SGPL1 (NM_003901.4), SMARCAL1 (NM_001127207.2), SYNPO (NM_001166208.2), TRPC6 (NM_004621.6), TTC21B (NM_024753.5), WDR19 (NM_025132.4), WDR73 (NM_032856.4), WT1 (NM_024426.6), XPO5 (NM_020750.3), ZMPSTE24 (NM_005857.5), FN1 (NM_212482.3), GLA (NM_000169.2), SIX1 (NM_005982.4).
